# An Evidence-Based Approach to the Use of Predictive Biomarkers in the Treatment of Non- Small Cell Lung Cancer (NSCLC)

**DOI:** 10.3390/cancers3033506

**Published:** 2011-09-13

**Authors:** Cindy Quinton, Peter M. Ellis

**Affiliations:** 1 Juravinski Cancer Centre, 699 Concession, St Hamilton, Hamilton, ON L8V 5C2, Canada; E-Mail: cquintongladstone@rogers.com; 2 Department of Oncology, McMaster University Hamilton, ON L8S 4L8, Canada

**Keywords:** Non-small-cell lung cancer, targeted therapy, epidermal growth factor receptor, tyrosine kinase inhibitor, molecular marker, biomarker

## Abstract

Recent advances in the treatment of non-small cell lung cancer (NSCLC) have led to improvements in patient survival and quality of life. It is unclear whether molecular abnormalities associated with NSCLC cell survival, growth and proliferation are useful in predicting treatment benefit. We conducted a systematic review to establish which biomarkers contribute meaningfully to the management of NSCLC. A team of researchers searched PubMed and conference proceedings (ASCO, ESMO, IASLC, USCAP) using MESH terms for NSCLC and randomized trials (RCT), plus keywords for variables of interest. Evidence from multiple RCTs confirmed that histologic subtype is prognostic for survival and predictive of treatment efficacy and/or toxicity in NSCLC. Likewise, activating mutations of the epidermal growth factor receptor (*EGFR*) are associated with benefit from EGFR tyrosine kinase inhibitors in patients with advanced non-squamous NSCLC and should be assessed routinely. No biomarkers to date reliably predict response to anti-Vascular Endothelial Growth Factor (VEGF) therapies. There are inconsistent data on the role of ERCC1, *BRCA*, Beta tubulin III, RRM1, *K-RAS*, or *TP-53* in treatment decisions. These tests should not be routinely used in selecting treatment at this time, whereas *EML4/ALK* translocations predict responses to specific targeted agents, the optimal assessment of this molecular abnormality has yet to be established. Personalized care of patients with NSCLC based on biomarkers is increasingly important to both clinical practice and research.

## 1. Introduction

Significant progress has been made in the treatment of non-small-cell lung cancer (NSCLC) over the last three decades. Initial approaches to the management of lung cancer administered identical combination chemotherapy, regardless of histology, to patients with NSCLC and small-cell lung cancer (SCLC) alike [1,2]. Subsequent research efforts have produced good evidence of both overall survival and quality of life improvements with first [3,4], second [5-7], and third-line [7] systemic therapies. Nonetheless, in spite of a wide array of both cytotoxic chemotherapies and newer molecularly targeted agents, treatment effects are modest at best. Median overall survival for the entire cohort of advanced NSCLC is still 10–12 months, although some molecularly defined subgroups appear to have a much more favorable prognosis [8]. NSCLC remains the leading cause of cancer death in the developing world.

Attempts to improve outcomes in NSCLC have resulted in strategies to individualize, or personalize treatment decisions, based on clinically or molecularly defined biomarkers, broadly defined as characteristics that are “objectively measured and evaluated as an indicator of normal biologic processes, pathogenic processes, or pharmacologic responses to a therapeutic intervention” [9]. The goal is to identify both prognostic factors, which correlate with a better (or worse) patient outcome independent of treatment, as well as predictive factors, which reliably indicate which patient subgroups will respond better (or worse) to treatment.

Potential predictive biomarkers include clinical characteristics, such as performance status, gender, race, and smoking status [7,8,10,11], as well as pathologic features such as histological subtypes of NSCLC [12,13]. More recently, translational researchers have identified molecular abnormalities which may predict for response and survival benefit from molecularly targeted agents, as well as standard chemotherapeutic agents. Gene expression profiling, using microarray-derived gene signatures to predict responses to individual drugs, is a promising technology which may soon be incorporated into treatment algorithms [14].

However, the rapid expansion of knowledge concerning biomarkers has created considerable uncertainty about their appropriate use in treatment decision making. Analytical and performance requirements of laboratory assays of biomarkers must be clearly stipulated to ensure validity, reliability, and reproducibility of findings. Data on tissue and patient sampling parameters must be carefully examined for potential sources of bias. Outcome measures in prospective randomized controlled trials must be re-evaluated to capture clinically relevant responses to treatment.

In this paper we will focus on the role of predictive biomarkers and their impact on decision-making in advanced NSCLC. We will review the key findings of randomized controlled trials in NSCLC evaluating biomarkers predictive of responses to anti-EGFR, anti-angiogenic, and chemo-therapeutic agents. Ideally the identification of biomarker-defined subgroups of patients for which there is a treatment by biomarker interaction will maximize the benefit to toxicity ratio for even moderately helpful therapy. These interactions include both quantitative effects, in which there are statistically significant differences in effect sizes between subgroups, and qualitative effects, in which the direction in effect size is opposite between subgroups.

### 1.1. Search Strategy and Selection Criteria

References for this review were found by searches of PubMed using the search terms: ‘Non-Small Cell Lung Cancer/drug therapy’ and ‘Randomised’ or ‘Randomized’ or ‘Randomly’ or ‘Trials’ or ‘Trial’ or ‘Study’, plus keywords for specific biomarkers. The most frequently occurring biomarkers were identified in meeting abstracts for the American Society of Clinical Oncology (ASCO), European Society of Medical Oncology (ESMO), and the International Association for the Study of Lung Cancer (IASLC) World Lung Congresses from 2005–2009. Review article bibliographies were also scanned for related publications. The articles selected were evaluated for clinical relevance using the following criteria: whether patient cohorts and treatments were clearly defined, whether the predictive impact of histology or biomarkers was well described, and whether outcomes of interest were ascertained on adequate follow-up. Only articles published in English from January 2005 to January 2011 were included.

## 2. Results and Discussion

### 2.1. Histology

There is consistent information from three randomized trials (Table 1) that histology is predictive of the benefit from pemetrexed, a multi-targeted anti-folate, which primarily inhibits thymidylate synthase (TS) [15]. In a subgroup analysis of the JMEI trial, patients with non-squamous histology treated second-line with pemetrexed had significantly longer survival than those who received docetaxel (HR = 0.77 9.3 *vs.* 8.0 months, p = 0.048) [16,17]. Likewise, survival in the JMDB first-line trial was significantly improved for those with adenocarcinoma receiving cisplatin/pemetrexed versus cisplatin/gemcitabine (12.6 *vs.* 10.4 months, HR = 0.84, CI = 0.71–0.98, p = 0.005). The outcomes were reversed for those with squamous histology, who benefitted more in the cisplatin/gemcitabine arm (10.8m *vs.* 9.4m for cisplatin/pemetrexed HR = 1.22, CI = 0.99–1.50, p = 0.05) [4]. For maintenance pemetrexed, there was a significant treatment by histology quantitative interaction whereby patients with nonsquamous histology had markedly longer progression free survival (PFS) and overall survival (OS) on maintenance therapy than did those with squamous tumours. (PFS HR = 0.44, 95% CI = 0.36–0.55, and OS HR = 0.70, 95% CI = 0.56–0.88, p = 0.002) [12]. This differential effect has been attributed to higher levels of TS expression in squamous cell malignancies [18,19]. However, one additional trial of carboplatin/pemetrexed versus carboplatin/gemcitabine did not show any interaction between histology and treatment effect [20]. Histology does not appear to be predictive of benefit from other cytotoxic agents [21].

Histology also appears predictive of treatment benefit for targeted agents. In both the BR.21 [7] and ISEL [11] trials evaluating an EGFR TKI versus best supportive care (BSC), higher objective response rates (ORR) were observed in patients with adenocarcinoma. Adenocarcinoma appeared predictive of better overall results in ISEL, but no significant interaction between histology and survival was observed in the BR.21 trial. No significant interaction effect was seen in the FLEX trial of cisplatin/vinorelbine +/- cetuximab, in which survival with all histological subtypes was improved by cetuximab [22,23].

Therapies targeted against vascular endothelial growth factor (VEGF) have demonstrated histology to be predictive for both efficacy and toxicity. In the ECOG 4599 [13] trial of carboplatin/paclitaxel +/− bevacizumab, patients with adenocarcinoma realized the greatest survival advantage. An earlier randomized phase II trial of the same agents [24] had shown an increased risk of fatal hemoptysis in patients receiving bevacizumab, which appeared to be associated with squamous histology. Life threatening hemorrhagic complications were not seen in other antiangiogenesis trials comparing vandetanib versus erlotinib [25], nor in the ESCAPE trial evaluating the addition of sorafenib to carboplatin and paclitaxel [26]. However patients with squamous histology in the ESCAPE trial randomized to chemotherapy plus sorafenib did appear to have an inferior overall survival.

Therefore, there is sufficient evidence demonstrating the value of histology as a predictive biomarker for NSCLC. Accordingly, accurate subclassification of NSCLC should be attempted in all lung cancer samples, highlighting the need to obtain adequate diagnostic material whenever possible. Immunohistochemical (IHC) staining favouring either adenocarcinoma (stains for TTF-1 and mucin, such as AB/PAS) or squamous cell carcinoma (such as CK5/6 and p63) should be routinely included in pathological assessment of small samples that cannot be accurately classified using conventional H&E staining.

### 2.2. Epidermal Growth Factor Receptor Status

The EGFR pathway has emerged as a preeminent target in NSCLC care. The transmembrane EGF receptor belonging to the ErbB family has an intracellular tyrosine kinase domain. It may be activated through ligand binding, as well as ligand-independently, through gene mutation and increased gene copy numbers. Once activated, the phosphorylation of multiple tyrosine kinases is triggered in a downstream cascade leading to cell proliferation, survival, angiogenesis and possibly migration. Due to the EGFR pathway's central role in the development and progression of cancer, agents inhibiting either the EGFR tyrosine kinase domain (gefitinib, erlotinib), or directed against the extracellular portion of the receptor (cetuximab, panitumumab) have been extensively tested in multiple cancer types. In NSCLC, researchers have debated which of several EGFR-related biomarkers would best identify patients likely to respond to these targeted therapies. As a result “a conflicting and confusing body of information now confronts practicing physicians” [27]. It is unclear whether (i) EGFR protein expression as measured by immunohistochemistry (IHC); (ii) *EGFR* gene copy number as evaluated by fluorescence *in-situ* hybridisation (FISH) or qRT-PCR; or (iii) gene mutations isolated by direct gene sequencing and other methods, should be employed in treatment algorithms.

#### 2.2.1. EGFR Protein Expression

IHC testing for EGFR protein expression has the advantage of wide availability in most pathology laboratories, and such a biomarker could be readily introduced into community clinical practice. Both the BR.21 [7] and ISEL [11] trials demonstrated that patients with EGFR overexpression by IHC had a higher ORR (BR.21 11% *vs.* 4%, p = 0.1, ISEL 8.2% *vs.* 1.5%, p value not reported) when treated with EGFR-TKIs, compared with those with EGFR IHC negative tumors. In both papers survival was extended by TKIs over placebo only in the patients with EGFR overexpression. The interaction of EGFR protein expression and treatment was significant for survival in the ISEL (p = 0.05), but not in BR.21 (p = 0.25).

On the other hand, the INTEREST trial of second-line gefitinib versus docetaxel [28], the BMS099 trial of carboplatin/paclitaxel +/− cetuximab [29], and the SATURN [30] trial of maintenance erlotinib versus placebo after platinum-based chemotherapy doublets, all failed to show differential outcomes (ORR, PFS, and OS) according to EGFR protein expression.

#### 2.2.2. EGFR Copy Number

The second and third-line trials of gefitinib and erlotinib reported significant correlations between increased *EGFR* gene copy number (by FISH) and extended overall survival with TKI treatment. However, the TRIBUTE [31], TALENT [32], and INTACT I/II [33-35] trials saw no improvement in OS for patients with *EGFR* amplification receiving an EGFR TKI. Furthermore, there was no association of increased gene copy number assessed by FISH, with survival in the INTEREST, SATURN, or BMS099 studies. In INTEREST a high *EGFR* copy number by FISH did identify those who would have a greater response to gefitinib as opposed to docetaxel [36].

#### 2.2.3. EGFR Mutations

Astute inquiry into the molecular underpinnings of the high response rate from EGFR TKIs seen among certain clinical groups such as females, never smokers, and those of Asian ethnicity, or with adenocarcinoma identified the presence of activating mutations of the EGFR TK domain [37-39]. The prevalence of such activating *EGFR* mutations may be as high as 40% in Asian populations, compared with only 10% in Caucasians [40]. Deletions in exon 19 and point mutations in at the L858R region of exon 21 have been associated with a more favourable prognosis and increased sensitivity to treatment with EGFR TKIs. A recent systematic review and meta-analysis of 80 articles concluded that *EGFR* mutations were predictive of response to single-agent TKIs (sensitivity = 0.78, 95% CI = 0.74–0.82, specificity = 0.86, 95% CI 0.82–0.89, positive likelihood ratio= 5.6, negative likelihood ratio = 0.25). Of note, increased EGFR gene copy number was also associated with response to TKIs, albeit with lower sensitivity and specificity [41].

In patients previously treated with chemotherapy, EGFR mutations predicted for increased ORR in the BR.21 (27% *vs.* 7%, p = 0.04) and ISEL (37.5% *vs.* 2.6%, p not reported) trials. Similarly, the SATURN trial of maintenance erlotinib versus placebo, reported a large gain in PFS for patients with an EGFR mutation treated with erlotinib (HR = 0.10, 95% CI = 0.04–0.25, p< 0.0001). However, no improvement in overall survival for mutation-positive patients treated with EGFR TKIs was observed in either the BR.21 or SATURN trials.

Trials comparing an EGFR TKI to chemotherapy, also demonstrate improved response rates and PFS for patients with *EGFR* mutations receiving an EGFR TKI. A qualitative interaction was demonstrated in the IPASS study contrasting first-line gefitinib with carboplatin/paclitaxel [8]. In those patients carrying *EGFR* mutations, PFS was significantly longer in the gefitinib arm (HR for progression or death = 0.48, 95% CI = 0.36–0.64, p < 0.001), whereas in those patients who were *EGFR* wild type, PFS was actually shorter with gefitinib than with standard chemotherapy (HR = 2.85, 95% CI = 2.05–3.98, p < 0.001). Analogous results were reported for the First Signal trial of gefitinib *versus* cisplatin/gemcitabine [42]. The INTEREST trial demonstrated increased ORR and PFS (HR = 0.16, 95% CI = 0.05 to 0.49, p = 0.001) for patients with *EGFR* mutation treated with gefitinib compared with docetaxel [36]. No significant difference in overall survival was demonstrated in these trials, or the West Japan Thoracic Oncology Group (WJTOG) trial of three cycles of carboplatin/paclitaxel followed by gefitinib or a further three cycles of chemotherapy [43]. It has been suggested that this discordance between PFS and OS is likely related to post study therapy, with crossover from the chemotherapy to the gefitinib arm at the time of progression [36].

Interestingly, the BMS099 trial evaluating the addition of the monoclonal antibody cetuximab to carboplatin and taxol found that *EGFR* mutation status was not predictive of cetuximab efficacy [29]. On the whole though, accumulated evidence suggests that *EGFR* mutation status is closely associated with the response in NSCLC to EGFR TKIs, especially in the first line setting. Diagnostic samples from patients with advanced NSCLC of nonsquamous subtype should be routinely assessed for known activating mutations of *EGFR* prior to the initiation of systemic therapies [44]. In addition, extrapolating from the IPASS results, EGFR TKIs should not be administered as first-line therapy for patients with wild-type *EGFR*.

#### 2.2.4. K-Ras Mutation Testing

The *K-Ras* gene encodes one of the intracellular signal transduction proteins in the EGFR pathway. Activating mutations at codons 12 and 13 in the GTPase domain of *K-RAS* occur in 15–25% of NSCLCs, almost exclusively in *EGFR* wild-type tumors. They are more common in patients with adenocarcinoma histology, former or current smokers and in Caucasians, rather than East Asians [39].

The presence of *K-RAS* mutations in other cancers such as adenocarcinoma of the colon is strongly predictive of a lack of benefit of EGFR therapy [45]. However, there is conflicting information about the predictive value of *RAS* mutations in NSCLC. In the FLEX and BMS099 trials, *K-RAS* mutation status was not associated with outcomes and in the SATURN trial *K-RAS* status was not predictive of overall survival [22,29]. However, a qualitative interaction was observed for *K-RAS* in the BR.21 trial [46]. The HR for OS in patients with *K-RAS* mutations was 1.67 (95% CI = 0.82-4.50, p = 0.31), whereas the HR in patients with wild-type *K-RAS* suggested benefit from erlotinib (HR = 0.69, 95% CI = 0.49–0.97, p = 0.03).

*K-RAS* status has also been reported to be associated with response to chemotherapy. In the JBR.10 trial evaluating adjuvant cisplatin/vinorelbine versus observation in completely resected stage IB/II NSCLC, patients with mutations in *H-RAS, K-RAS* and *N-RAS* did not seem to benefit from adjuvant chemotherapy (HR = 0.95, p = 0.87). Patients with wild-type *RAS* did experience a treatment related survival benefit (HR = 0.69, p = 0.03), but there was no significant interaction effect for treatment by *RAS* mutation status (p = 0.29) [47]. Based on the inconclusive data, routine testing for *RAS* mutations is not recommended.

### 2.3. Biomarkers of Response to Chemotherapy

Until recently, platinum-based cytotoxic chemotherapy has represented the standard of care for unselected patients with advanced NSCLC. Platinum doublets with either gemcitabine or taxanes, including paclitaxel and docetaxel, are extensively used for treatment. However response rates as low as 19% have been reported in at least one large trial [48] and side effects of therapy may impair quality of life. While the helpful role of histology in the prospective selection of individuals most likely to benefit from therapy has been described above, its limitations drive the search for further markers of chemosensitivity.

#### 2.3.1. Cisplatin

Cisplatin exerts its antiproliferative effects primarily via DNA damage, through the formation of DNA adducts, which hamper DNA polymerase activity, ultimately leading to apoptosis. As such, molecular components of DNA repair mechanisms constitute attractive candidates for biomarker researchers.

In the IALT-Bio analysis, high levels of expression of the excision repair cross-complementation group 1 enzyme (ERCC1) as measured by IHC were prognostic for improved OS in the placebo group (HR = 0.66, 95% CI = 0.4–0.90, p = 0.009) while low levels predicted benefit from chemotherapy (HR = 0.65, 95% CI = 0.50–0.86, p = 0.002) [49]. As this enzyme plays a key role in the nucleotide excision repair pathway, its absence may affect the ability of the cells to repair DNA damage induced by platinum agents. Both ERCC1 protein expression as assessed by in situ technology and ERCC1 polymorphisms are under investigation as predictors of response, but results are conflicting [10,50]. The MADeIT study, a prospective phase II trial of first-line treatment of advanced NSCLC determined by ERCC1 and RRM1 levels, reported an ORR of 44% and one year survival of 59% [51]. Further prospective validation of ERCC1 may be provided by the International Tailored Chemotherapy Adjuvant Trial (ITACA) as patients are randomised to standard adjuvant platinum-based chemotherapy or to treatment personalized on the basis of quantitative ERCC1 and TS gene expression.

The *BRCA1* enzyme also plays an important role in response to DNA damage. One small NSCLC trial by the Spanish Lung Cancer Group has suggested that patients with a high *BRCA1* expression level as measured by qRT-PCR could safely omit platinum from adjuvant chemotherapy, but these findings have not been confirmed [52]. Further prospective validation trials of similar genomic-driven approaches are required.

#### 2.3.2. Gemcitabine

Gemcitabine acts as a specific pyrimidine nucleoside antimetabolite whose active metabolite blocks the activity of ribonucleotide reductase (RR), an enzyme essential for the production of deoxyribonucleotides. The RRM1 subunit of RR as measured by *in situ* methods may predict response to gemcitabine [53]. In one analysis of 81 advanced NSCLC patients, low levels of both RRM1 and ERCC1 (m)RNA by qRT-PCR were associated with a significant increase in survival with gemcitabine/cisplatin treatment relative to other chemotherapy regimens [54]. These results require prospective validation. Outcomes of gemcitabine treatment have also reportedly been correlated with RRM1 polymorphisms [55]. Other molecular predictors of gemcitabine sensitivity, such as low levels of expression of 5'-nucleotidase protein, which might be responsible for early inactivation of the drug, remain investigational.

#### 2.3.3. Taxanes

Both paclitaxel and docetaxel bind to β-tubulin to stabilize microtubules, inhibiting their depolymerisation, thus interfering with spindle assembly, and leading to mitotic arrest. Their target, β-tubulin, has shown promise as a biomarker in advanced NSCLC [56]. Retrospective studies provide support for a significant positive predictive role of low levels of class III β-tubulin for ORR, PFS, and OS in patients treated with taxane-based chemotherapy [56,57]. While the JBR.10 trial found class III β-tubulin to be predictive of benefit from adjuvant chemotherapy, the LACE-BIO meta-analysis did not confirm these findings [58].

### 2.4. Biomarkers of Response to Anti-Angiogenic Agents

Biomarker analyses of studies evaluating angiogenesis inhibitors have yet to identify consistent predictive biomarkers. Histological subtype is considered a predictor of toxicity from bevacizumab. Other analyses have been exploratory and require prospective validation. In the ECOG 4599 trial evaluating the addition of bevacizumab to carboplatin/paclitaxel low baseline intracellular adhesion molecule levels (ICAM) were associated with improved ORR (32% *vs.* 14%, p = 0.02), OS (P = 0.00005) and 1 year survival (65% *vs.* 25%) relative to high ICAM, regardless of treatment arm. High serum VEGF levels predicted ORR, but not a survival advantage with chemotherapy/bevacizumab [59].

The AVAiL investigators reported that higher baseline levels of ICAM, vascular cell adhesion molecule (VCAM), Beta fibroblast growth factor (Beta FGF) and VEGF were all correlated with shorter OS [60]. The ZODIAC trial randomized patients to second-line docetaxel +/− vandetanib, a multitargeted inhibitor of EGFR, VEGFR, and RET [61,62]. Multiple biologic markers, including EGFR by both IHC and FISH, KRAS mutations, VEGF and VEGF-2 levels, failed to predict outcomes, possibly due to the low number of tissue samples submitted for correlative biomarker studies.

The development of hypertension has been suggested as a biomarker of efficacy for antiangiogenesis therapy. In the ECOG 4599 trial, the development of hypertension during treatment was suggestive of improved outcomes for patients receiving bevacizumab [63]. However, this was not predictive of differential outcomes with cediranib in an analysis of data from BR.24, a placebo-controlled phase II trial of carboplatin/paclitaxel chemotherapy +/− this VEGFR TKI [64].

### 2.5. Novel Molecular Markers

Other biomarkers on the horizon include cell cycle regulators such as the tumor-suppressor gene *TP53*. In the JBR.10 adjuvant chemotherapy trial, only those patients with P53 expression as measured by nuclear staining derived significant effects from chemotherapy [65]. The interaction for chemotherapy and p53 protein was significant (p = 0.02). Mutation testing confirmed that only those with wild-type *TP53* benefitted from therapy.

The recently discovered translocation of the echinoderm microtubule-associated protein-like 4/anaplastic lymphoma kinase (*EML4/ALK*) fusion oncogene, shows great promise as a therapeutic target for NSCLC. This translocation, which is mutually exclusive with *EGFR* and *KRAS* mutations, is seen in 1.6 to 6.7% of NSCLC patients, most often in non-smokers and those with adenocarcinomas [66]. It may represent yet another mechanism of resistance to EGFR TKIs, through constitutive ALK signaling. The presence of the *EML4/ALK* translocation appears strongly predictive of benefit from crizotinib, a dual tyrosine kinase inhibitor of c-MET and the ALK receptor. A recently published phase I/II trial of crizotinib in patients with tumors harbouring an *ALK* rearrangement by FISH, demonstrated a 57% ORR and a disease control rate of 87% at 8 weeks [67]. Phase III trials of this agent are ongoing in both first and second-line therapy of NSCLC.

Many other biomarkers are under evaluation and while promising *in vitro*, most lack prospective clinical validation. In the BR21 trial, TGF-alpha was found to predict lack of benefit for erlotinib, although this should be considered hypothesis generating rather than guiding treatment selection at this time [68]. The insulin-like growth factor receptor-1 (IGFR-1) is a transmembrane protein encoded by an oncogene on chromosome 15q25–q26. Early trials of the IGFR-1 antibody figitumumab have demonstrated a differential response in squamous histology NSCLC, which may overexpress IGF-1R [69]. While the results from the phase III trial of the carboplatin/paclitaxel +/− figitumumab showed no overall survival benefit, patients with the highest baseline levels of free serum IGF appeared to show some benefit [70]. These data require confirmation in further trials. Gene amplification of MET, the transmembrane tyrosine kinase receptor for hepatocyte growth factor (HGF) may lead to primary resistance to EGFR TKI's through enhanced activation of the AKT pathway. Increased expression of the MET ligand HGF has also been implicated in this process [71]. Recent data from a randomized phase II trial of erlotinib with or without MetMab (monoclonal antibody to MET) suggested that the addition of MetMab to erlotinib improved PFS and OS in patients who are Met diagnostic positive (moderate or intense expression of MET in 50% or more of cells) [72]. Other biomarkers associated with intrinsic and/or acquired resistance to EGFR TKIs include rare EGFR mutations, HER2 and 3 and their TK domain mutations [73] and PTEN loss.

### 2.6. Gene Expression Profiling

This year, total plasma DNA levels [74], XPC gene intron polymorphisms [75], and plasma nucleosome levels [75] have all been reported to correlate with response to chemotherapy. Research is also ongoing to identify predictive gene signatures. A five-gene signature was reported by Chen and colleagues as prognostic for survival with NSCLC [76]. However, it was derived from, and validated in, tumor samples from patients who had not received chemotherapy and so does not provide predictive information. A tissue microarray analysis of samples from 133 patients enrolled in the control arm of the JBR.10 trial led to the derivation of a 15-gene signature which correlated well with survival. The gene signature also appeared predictive for survival, as patients at “high risk” for recurrence as defined by the signature has significantly improved survival from adjuvant cisplatin/vinorelbine (HR = 0.33, 95% CI = 0.17–0.63, p = 0.0005). Moreover chemotherapy was associated with a shorter OS in “low risk” individuals (p = 3.67, 95% CI = 1.22–11.06, p = 0.0133), producing a highly significant qualitative interaction term. (p = 0.0001) [14].

## 3. Conclusions

Lung oncologists have seen an explosion in potential treatments in recent years. These advances have been met with great enthusiasm, as patients and physicians alike strive to take advantage of all opportunities. However, many molecularly targeted agents have not shown improvements in patient outcomes when tested in unselected patient populations. According to Govindan, the era of using molecularly targeted therapies in unselected populations is drawing to a close [27]. However, the available evidence summarized in this review would suggest that the only biomarkers that should routinely be incorporated into current practice are histologic subtyping and EGFR mutation status. Evidence for other biomarkers such as RAS, EGFR protein expression and gene copy number, as well as predictors of chemotherapy sensitivity such as ERCC1, RRM 1, p53 and beta tubulin levels, is inconsistent and requires further evaluation.

The incorporation of translational research in the design of ongoing clinical trials is an important and yet challenging area of research. It is essential to aid in the identification of patient subgroups most likely to benefit from newer biologic agents and from cytotoxic chemotherapies used in novel ways. Increased data are needed to reduce potential sampling bias. Nonetheless many challenges exist including the availability of adequate tissue samples and willingness of patients to consent to translational studies. One approach to consider is to make blood and tissue specimen collection a mandatory component for enrolment of patients onto a clinical trial. Such a strategy was adopted in the MARVEL trial of second line erlotinib *versus* chemotherapy. This trial promised important knowledge about the relative outcomes of chemotherapy *versus* a targeted agent in EGFR FISH positive and negative subgroups. However, the trial closed prematurely because of poor recruitment.

There is a need for standardized methodology in biomarker research. Biomarkers must be prospectively validated and then evaluated for reproducibility in clinical trials. Consistent methodology, definition of relevant endpoints, and standardized reporting may clarify many of the conflicting data reviewed above. Relevant guidelines for the development and incorporation of biomarker studies in early clinical trials of novel agents have been outlined by the Biomarkers Task Force of the National Cancer Institute (NCI) Investigational Drug Steering Committee. Definitions of nomenclature, analytical and performance requirements of laboratory assays, statistical considerations arising from sequential analyses of multiple variables, as well as recommendations for the sponsor and investigator in new therapeutic studies are all provided [9].

The availability of tissue, the possibility of tumor heterogeneity and the potential for molecular phenotypes to change over time and in response to prior therapy all pose challenges to the use of biomarker data. The use of archival tissue, either paraffin embedded or fresh frozen tissue may not be representative of the surviving clones [27]. There is potential for the molecular profile of NSCLC tumors to change at the time of metastasis, as has been demonstrated in breast cancer cases [77]. This possibility has been explored by Cortot's group, who examined K-RAS and EGFR mutation status with PCR and direct sequencing on 21 primary tumors and their associated metastases. There were no EGFR mutations detected and discordant for K-RAS mutation status was observed in only three patients [78]. Further research is needed in this area.

Many researchers are looking to circulating tumor cells in peripheral blood as an alternative method of biomarker assessment rather than analyzing pathological specimens. One example from Dutch trialists, assessed serum levels of transforming growth factor-alpha (TGFa), amphiregulin (ARG), and insulin-like growth factor (IGF) which have all been associated with resistance to EGFR-TKIs. Results suggest that serum concentrations of TGFa and ARG were predictive of EGFR-TKI responses [79]. This approach requires further validation.

The potential implications of biomarker testing for pathology departmental infrastructure, training, and costs could be considerable. Demands for more substantial tissue samples in NSCLC to support such intensive investigations will no doubt strain limited imaging, endoscopic, and surgical resources. Additionally, the emergence of resistance to a molecularly targeted agent may correlate with changing molecular profile of the tumor. This could result in a clinical environment in which repeat biopsies are required upon disease progression, to re-evaluate the molecular expression of the tumor. Fortunately a tailored strategy promises many rewards, not the least of which is increased cost-effectiveness. The identification of clinical or molecular biomarkers of response and/or toxicity may ensure that only those patients most likely to benefit will be offered costly and perhaps only moderately effective treatment [79,80]. It is uncertain whether biomarker-driven approaches to treatment selection produce superior outcomes to those selected on the basis of empiric algorithms. Clinical trials such as the MD Anderson Cancer Centre's BATTLE phase II study, which randomizes patients to second line treatment based on 11 biomarkers including EGFR, KRAS, BRAF, Cyclin D1, VEGF, VEGFR-2, and RXR will no doubt explore the feasibility of the “personalized medicine” approach to the management of NSCLC [81,82].

## Figures and Tables

**Table 1.** Evidence for Biomarker Use in Advanced NSCLC.

**Trials**	**Number of patients**	**Findings**
*Histology*
7 trials – E4599 plus randomized phase II trial, BR21, SWOG database analysis (S9806, S0003, S9308), JMEI, JMBD, JMEN	5408	Adenocarcinoma histology prognostic for ↑OS. Adenocarcinoma predictive of ↑ PFS and OS for bevacizumab. Squamous ca predictive of toxicity from bevacizumab. Adenocarcinoma predictive of ↑ ORR, but not OS for erlotinib. Non-squamous histology predictive of ↑ORR, PFS and OS for pemetrexed
*EGFR protein expression (IHC)*		
6 trials – BR21, ISEL, IPASS, INTEREST, SATURN, TALENT	2691	EGFR protein expression predictive of ↑ORR and OS in BR21 and ISEL, but not predictive of TTP or OS in other studies
*EGFR copy number (FISH or qPCR)*		
9 trials – BR21, ISEL, IPASS, INTEREST, SATURN, TALENT, TRIBUTE, INTACT I/II	2994	Some evidence that EGFR gene copy number prognostic for ↓ OS EGFR gene copy number predictive of ↑OS in BR21 and ISEL, but not predictive of PFS or OS in other trials
*EGFR mutation*		
14 trials – BR21, ISEL, SATURN, IPASS, First Signal, WJTOG3405, NEJ002, INTEREST, IDEAL I/II, INTACT I/II, TALENT, TRIBUTE	3259	EGFR gene mutations prognostic for ↑ OS. EGFR mutations predictive of ↑ ORR, but not OS in 2nd/3rd line therapy. In studies comparing EGFR TKI to chemotherapy, EGFR mutations predictive of ↑ ORR and PFS. OS data are immature
*K-Ras mutations*		
8 trials	2442	Mixed information re prognostic value of K-Ras mutations. Likely weakly prognostic for ↓ OS. BR10 suggests K-Ras mutations predict lack of OS benefit from adjuvant chemotherapy. Two studies suggestive K-Ras predictive of ↓ OS for patients on EGFR TKI, while 4 trials show K-Ras not predictive of ORR, PFS or OS differences

## References

[1] Hansen H.H., Selawry O.S., Simon R., Carr D.T., van Wyk C.E., Tucker R.D., Sealy R. (1976). Combination chemotherapy of advanced lung cancer: A randomized trial. Cancer.

[2] Pao W., Chmielecki J. (2010). Rational, biologically based treatment of egfr-mutant non-small-cell lung cancer. Nat. Rev. Cancer.

[3] (1995). Anonymous Chemotherapy in non-small cell lung cancer: A meta-analysis using updated data on individual patients from 52 randomised clinical trials. Non-small cell lung cancer collaborative group. Br. Med. J..

[4] Scagliotti G.V., Parikh P., von Pawel J., Biesma B., Vansteenkiste J., Manegold C., Serwatowski P., Gatzemeier U., Digumarti R., Zukin M. (2008). Phase iii study comparing cisplatin plus gemcitabine with cisplatin plus pemetrexed in chemotherapy-naive patients with advanced-stage non-small-cell lung cancer. J. Clin. Oncol..

[5] Fossella F.V., DeVore R., Kerr R.N., Crawford J., Natale R.R., Dunphy F., Kalman L., Miller V., Lee J.S., Moore M. (2000). Randomized phase iii trial of docetaxel versus vinorelbine or ifosfamide in patients with advanced non-small-cell lung cancer previously treated with platinum-containing chemotherapy regimens. The tax 320 non-small cell lung cancer study group. J. Clin. Oncol..

[6] Shepherd F.A., Dancey J., Ramlau R., Mattson K., Gralla R., O'Rourke M., Levitan N., Gressot L., Vincent M., Burkes R. (2000). Prospective randomized trial of docetaxel versus best supportive care in patients with non-small-cell lung cancer previously treated with platinum-based chemotherapy. J. Clin. Oncol..

[7] Shepherd F.A., Rodrigues Pereira J., Ciuleanu T., Tan E.H., Hirsh V., Thongprasert S., Campos D., Maoleekoonpiroj S., Smylie M., Martins R. (2005). Erlotinib in previously treated non-small-cell lung cancer. N. Engl. J. Med..

[8] Mok T.S., Wu Y.L., Thongprasert S., Yang C.H., Chu D.T., Saijo N., Sunpaweravong P., Han B., Margono B., Ichinose Y. (2009). Gefitinib or carboplatin-paclitaxel in pulmonary adenocarcinoma. N. Engl. J. Med..

[9] Dancey J.E., Dobbin K.K., Groshen S., Jessup J.M., Hruszkewycz A.H., Koehler M., Parchment R., Ratain M.J., Shankar L.K., Stadler W.M. (2010). Guidelines for the development and incorporation of biomarker studies in early clinical trials of novel agents. Clin. Cancer Res..

[10] Park S.Y., Hong Y.C., Kim J.H., Kwak S.M., Cho J.H., Lee H.L., Ryu J.S. (2006). Effect of ercc1 polymorphisms and the modification by smoking on the survival of non-small cell lung cancer patients. Med. Oncol..

[11] Thatcher N., Chang A., Parikh P., Rodrigues Pereira J., Ciuleanu T., von Pawel J., Thongprasert S., Tan E.H., Pemberton K., Archer V. (2005). Gefitinib plus best supportive care in previously treated patients with refractory advanced non-small-cell lung cancer: Results from a randomised, placebo-controlled, multicentre study (iressa survival evaluation in lung cancer). Lancet.

[12] Ciuleanu T., Brodowicz T., Zielinski C., Kim J.H., Krzakowski M., Laack E., Wu Y.L., Bover I., Begbie S., Tzekova V. (2009). Maintenance pemetrexed plus best supportive care versus placebo plus best supportive care for non-small-cell lung cancer: A randomised, double-blind, phase 3 study. Lancet.

[13] Sandler A., Gray R., Perry M.C., Brahmer J., Schiller J.H., Dowlati A., Lilenbaum R., Johnson D.H. (2006). Paclitaxel-carboplatin alone or with bevacizumab for non-small-cell lung cancer. N. Engl. J. Med..

[14] Zhu C.Q., Ding K., Strumpf D., Weir B.A., Meyerson M., Pennell N., Thomas R.K., Naoki K., Ladd-Acosta C., Liu N. (2010). Prognostic and predictive gene signature for adjuvant chemotherapy in resected non-small-cell lung cancer. J. Clin. Oncol..

[15] Saloura V., Wang L., Schwed D., Vachani A., Langer C.J., Albelda S. (2010). Personalizing treatment with pemetrexed (pem) in patients with malignant mesothelioma (mpm) and non-small cell lung cancer (nsclc): The role of thimidylate synthase. ASCO Meet. Abstr..

[16] Hanna N., Shepherd F.A., Fossella F.V., Pereira J.R., de Marinis F., von Pawel J., Gatzemeier U., Tsao T.C., Pless M., Muller T. (2004). Randomized phase iii trial of pemetrexed versus docetaxel in patients with non-small-cell lung cancer previously treated with chemotherapy. J. Clin. Oncol..

[17] Peterson P., Park K., Fossella F., Gatzemeier U., John W., Scagliotti G.V. (2007). Is pemetrexed more effective in adenocarcinoma and large cell lung cancer than in squamous cell carcinoma? A retrospective analysis of a phase iii trial of pemetrexed vs. docetaxel in previously treated patients with advanced non-small cell lung cancer (nsclc). J. Thorac. Oncol..

[18] Ceppi P., Volante M., Saviozzi S., Rapa I., Novello S., Cambieri A., Lo Iacono M., Cappia S., Papotti M., Scagliotti G.V. (2006). Squamous cell carcinoma of the lung compared with other histotypes shows higher messenger rna and protein levels for thymidylate synthase. Cancer.

[19] Scagliotti G., Hanna N., Fossella F., Sugarman K., Blatter J., Peterson P., Simms L., Shepherd F.A. (2009). The differential efficacy of pemetrexed according to nsclc histology: A review of two phase iii studies. Oncologist.

[20] Grønberg B.H., Bremnes R.M., Fløtten Ø, Amundsen T., Brunsvig P.F., Hjelde H.H., Kaasa S., von Plessen C., Stornes F., Tollåli T. (2009). Phase iii study by the norwegian lung cancer study group: Pemetrexed plus carboplatin compared with gemcitabine plus carboplatin as first-line chemotherapy in advanced non–small-cell lung cancer. J. Clin. Oncol..

[21] Chansky K., Mack P.C., Crowley J.J., Lara P.N., Hirsch F.R., Franklin W.A., Gandara D.R. (2009). Chemotherapy outcomes by histologic subtype of non-small cell lung cancer (NSCLC): Analysis of the swog database for antimicrotubule-platinum therapy. J. Thorac. Oncol..

[22] O'Byrne K.J., Bondarenko I., Barrios C., Eschbach C., Martens U., Hotko Y., Kortsik C., Celik I., Stroh C., Pirker R. (2009). Molecular and clinical predictors of outcome for cetuximab in non-small cell lung cancer (nsclc): Data from the flex study. J. Clin. Oncol..

[23] Pirker R., Pereira J.R., Szczesna A., von Pawel J., Krzakowski M., Ramlau R., Vynnychenko I., Park K., Yu C.T., Ganul V. (2009). Cetuximab plus chemotherapy in patients with advanced non-small-cell lung cancer (flex): An open-label randomised phase iii trial. Lancet.

[24] Johnson D.H., Fehrenbacher L., Novotny W.F., Herbst R.S., Nemunaitis J.J., Jablons D.M., Langer C.J., DeVore R.F., Gaudreault J., Damico L.A. (2004). Randomized phase ii trial comparing bevacizumab plus carboplatin and paclitaxel with carboplatin and paclitaxel alone in previously untreated locally advanced or metastatic non-small-cell lung cancer. J. Clin. Oncol..

[25] Natale R.B., Bodkin D., Govindan R., Sleckman B.G., Rizvi N.A., Capo A., Germonpre P., Eberhardt W.E., Stockman P.K., Kennedy S.J. (2009). Vandetanib versus gefitinib in patients with advanced non-small-cell lung cancer: Results from a two-part, double-blind, randomized phase ii study. J. Clin. Oncol..

[26] Scagliotti G., Novello S., von Pawel J., Reck M., Pereira J.R., Thomas M., Abrao Miziara J.E., Balint B., De Marinis F., Keller A. (2010). Phase iii study of carboplatin and paclitaxel alone or with sorafenib in advanced non-small-cell lung cancer. J. Clin. Oncol..

[27] Govindan R. (2010). Interesting biomarker to select ideal patients for epidermal growth factor receptor tyrosine kinase inhibitors: Yes, for egfr mutation analysis, others, i pass. J. Clin. Oncol..

[28] Douillard J.-Y., Kim E., Hirsh V., Mok T., Socinski M., Gervais R., Wu Y., Li L., Sellers M., Lowe E. (2007). Gefitinib (iressa) versus docetaxel in patients with locally advanced or metastatic non-small-cell lung cancer pre-treated with platinum-based chemotherapy: A randomized, open-label phase iii study (interest). J. Thorac. Oncol..

[29] Khambata-Ford S., Harbison C.T., Hart L.L., Awad M., Xu L.A., Horak C.E., Dakhil S., Hermann R.C., Lynch T.J., Weber M.R. (2010). Analysis of potential predictive markers of cetuximab benefit in bms099, a phase iii study of cetuximab and first-line taxane/carboplatin in advanced non-small-cell lung cancer. J. Clin. Oncol..

[30] Cappuzzo F., Ciuleanu T., Stelmakh L., Cicenas S., Szczesna A., Juhasz E., Esteban E., Molinier O., Brugger W., Melezinek I. (2010). Erlotinib as maintenance treatment in advanced non-small-cell lung cancer: A multicentre, randomised, placebo-controlled phase 3 study. Lancet Oncol..

[31] Herbst R.S., Prager D., Hermann R., Fehrenbacher L., Johnson B.E., Sandler A., Kris M.G., Tran H.T., Klein P., Li X. (2005). Tribute: A phase iii trial of erlotinib hydrochloride (osi-774) combined with carboplatin and paclitaxel chemotherapy in advanced non-small-cell lung cancer. J. Clin. Oncol..

[32] Gatzemeier U., Pluzanska A., Szczesna A., Kaukel E., Roubec J., de Rosa F., Milanowski J., Karnicka-Mlodkowski H., Pesek M., Serwatowski P. (2007). Phase iii study of erlotinib in combination with cisplatin and gemcitabine in advanced non-small-cell lung cancer: The tarceva lung cancer investigation trial. J. Clin. Oncol..

[33] Bell D.W., Lynch T.J., Haserlat S.M., Harris P.L., Okimoto R.A., Brannigan B.W., Sgroi D.C., Muir B., Riemenschneider M.J., Iacona R.B. (2005). Epidermal growth factor receptor mutations and gene amplification in non-small-cell lung cancer: Molecular analysis of the ideal/intact gefitinib trials. J. Clin. Oncol..

[34] Giaccone G., Herbst R.S., Manegold C., Scagliotti G., Rosell R., Miller V., Natale R.B., Schiller J.H., von Pawel J., Pluzanska A. (2004). Gefitinib in combination with gemcitabine and cisplatin in advanced non–small-cell lung cancer: A phase iii trial—Intact 1. J. Clin. Oncol..

[35] Herbst R.S., Giaccone G., Schiller J.H., Natale R.B., Miller V., Manegold C., Scagliotti G., Rosell R., Oliff I., Reeves J.A. (2004). Gefitinib in combination with paclitaxel and carboplatin in advanced non-small-cell lung cancer: A phase iii trial—Intact 2. J. Clin. Oncol..

[36] Douillard J.Y., Shepherd F.A., Hirsh V., Mok T., Socinski M.A., Gervais R., Liao M.L., Bischoff H., Reck M., Sellers M.V. (2010). Molecular predictors of outcome with gefitinib and docetaxel in previously treated non-small-cell lung cancer: Data from the randomized phase iii interest trial. J. Clin. Oncol..

[37] Lynch T.J., Bell D.W., Sordella R., Gurubhagavatula S., Okimoto R.A., Brannigan B.W., Harris P.L., Haserlat S.M., Supko J.G., Haluska F.G. (2004). Activating mutations in the epidermal growth factor receptor underlying responsiveness of non–small-cell lung cancer to gefitinib. N. Engl. J. Med..

[38] Paez J.G., Janne P.A., Lee J.C., Tracy S., Greulich H., Gabriel S., Herman P., Kaye F.J., Lindeman N., Boggon T.J. (2004). Egfr mutations in lung cancer: Correlation with clinical response to gefitinib therapy. Science.

[39] Pao W., Miller V., Zakowski M., Doherty J., Politi K., Sarkaria I., Singh B., Heelan R., Rusch V., Fulton L. (2004). Egf receptor gene mutations are common in lung cancers from “never smokers” and are associated with sensitivity of tumors to gefitinib and erlotinib. Proc. Natl. Acad. Sci. USA.

[40] Coate L.E., John T., Tsao M.S., Shepherd F.A. (2009). Molecular predictive and prognostic markers in non-small-cell lung cancer. Lancet Oncol..

[41] Dahabreh I.J., Linardou H., Siannis F., Kosmidis P., Bafaloukos D., Murray S. (2010). Somatic egfr mutation and gene copy gain as predictive biomarkers for response to tyrosine kinase inhibitors in non-small cell lung cancer. Clin. Cancer Res..

[42] Lee J.S., Park K., Kim S.W., Lee D.H., Kim H.T., Han J.Y., Yun T., Ahn M.J., Ahn J.S., Suh C. (2009). A randomized phase iii study of gefitinib (iressatm) versus standard chemotherapy (gemcitabine plus cisplatin) as a first-line treatment for never-smokers with advanced or metastatic adenocarcinoma of the lung. J. Thorac. Oncol..

[43] Takeda K., Hida T., Sato T., Ando M., Seto T., Satouchi M., Ichinose Y., Katakami N., Yamamoto N., Kudoh S. (2010). Randomized phase iii trial of platinum-doublet chemotherapy followed by gefitinib compared with continued platinum-doublet chemotherapy in japanese patients with advanced non-small-cell lung cancer: Results of a west japan thoracic oncology group trial (wjtog0203). J. Clin. Oncol..

[44] Ogita S., Wozniak A.J. (2010). Refining the treatment of advanced nonsmall cell lung cancer. Lung Cancer.

[45] Van Cutsem E., Kohne C.H., Hitre E., Zaluski J., Chang Chien C.R., Makhson A., D'Haens G., Pinter T., Lim R., Bodoky G. (2009). Cetuximab and chemotherapy as initial treatment for metastatic colorectal cancer. N. Engl. J. Med..

[46] Zhu C.Q., da Cunha Santos G., Ding K., Sakurada A., Cutz J.C., Liu N., Zhang T., Marrano P., Whitehead M., Squire J.A. (2008). Role of kras and egfr as biomarkers of response to erlotinib in national cancer institute of canada clinical trials group study br.21. J. Clin. Oncol..

[47] Winton T., Livingston R., Johnson D., Rigas J., Johnston M., Butts C., Cormier Y., Goss G., Inculet R., Vallieres E. (2005). Vinorelbine plus cisplatin vs. Observation in resected non-small-cell lung cancer. N. Engl. J. Med..

[48] Schiller J.H., Harrington D., Belani C.P., Langer C., Sandler A., Krook J., Zhu J., Johnson D.H. (2002). The Eastern Cooperative Oncology G. Comparison of four chemotherapy regimens for advanced non-small-cell lung cancer. N. Engl. J. Med..

[49] Olaussen K.A., Dunant A., Fouret P., Brambilla E., Andre F., Haddad V., Taranchon E., Filipits M., Pirker R., Popper H.H. (2006). DNA repair by ercc1 in non-small-cell lung cancer and cisplatin-based adjuvant chemotherapy. N. Engl. J. Med..

[50] Toschi L., Cappuzzo F. (2010). Impact of biomarkers on non-small cell lung cancer treatment. Target. Oncol..

[51] Simon G., Sharma A., Li X., Hazelton T., Walsh F., Williams C., Chiappori A., Haura E., Tanvetyanon T., Antonia S. (2007). Feasibility and efficacy of molecular analysis-directed individualized therapy in advanced non-small-cell lung cancer. J. Clin. Oncol..

[52] Rosell R., Skrzypski M., Jassem E., Taron M., Bartolucci R., Sanchez J.J., Mendez P., Chaib I., Perez-Roca L., Szymanowska A. (2007). Brca1: A novel prognostic factor in resected non-small-cell lung cancer. PLoS One.

[53] Reynolds C., Obasaju C., Schell M.J., Li X., Zheng Z., Boulware D., Caton J.R., Demarco L.C., O'Rourke M.A., Shaw Wright G. (2009). Randomized phase iii trial of gemcitabine-based chemotherapy with in situ rrm1 and ercc1 protein levels for response prediction in non-small-cell lung cancer. J. Clin. Oncol..

[54] Rosell R., Taron M., Alberola V., Massuti B., Felip E. (2003). Genetic testing for chemotherapy in non-small cell lung cancer. Lung Cancer.

[55] Bepler G., Zheng Z., Gautam A., Sharma S., Cantor A., Sharma A., Cress W.D., Kim Y.C., Rosell R., McBride C. (2005). Ribonucleotide reductase m1 gene promoter activity, polymorphisms, population frequencies, and clinical relevance. Lung Cancer.

[56] Seve P., Reiman T., Dumontet C. (2010). The role of betaiii tubulin in predicting chemoresistance in non-small cell lung cancer. Lung Cancer.

[57] Kang C.H., Jang B.G., Kim D.W., Chung D.H., Kim Y.T., Jheon S., Sung S.W., Kim J.H. (2010). The prognostic significance of ercc1, brca1, xrcc1, and betaiii-tubulin expression in patients with non-small cell lung cancer treated by platinum- and taxane-based neoadjuvant chemotherapy and surgical resection. Lung Cancer.

[58] Reiman T., Seve P., Veillard A.S., Soria J.C., Rosell R., Taron M., Graziano S., Kratzke R., Seymour L., Shepherd F. (2009). Validation of the prognostic and predictive value of class iii beta tubulin (tubb3) for adjuvant chemotherapy (act) in completely resected non-small cell lung cancer (r-nsclc): Results from four randomized trials. J. Thorac. Oncol..

[59] Dowlati A., Gray R., Sandler A.B., Schiller J.H., Johnson D.H. (2008). Cell adhesion molecules, vascular endothelial growth factor, and basic fibroblast growth factor in patients with non-small cell lung cancer treated with chemotherapy with or without bevacizumab—An eastern cooperative oncology group study. Clin. Cancer Res..

[60] Leighl N., Reck M., de Haas S., Evers S., Delmar P., Manegold C., Scherer S. (2009). Analysis of biomarkers (bms) in the avail phase iii randomised study of first-line bevacizumab (bv) with cisplatin-gemcitabine (cg) in patients (pts) with non-small cell lung cancer (NSCLC). Eur. J. Cancer Suppl..

[61] Hanrahan E.O., Lin H.Y., Kim E.S., Yan S., Du D.Z., McKee K.S., Tran H.T., Lee J.J., Ryan A.J., Langmuir P. (2010). Distinct patterns of cytokine and angiogenic factor modulation and markers of benefit for vandetanib and/or chemotherapy in patients with non-small-cell lung cancer. J. Clin. Oncol..

[62] Herbst R.S., Sun Y., Eberhardt W.E., Germonpre P., Saijo N., Zhou C., Wang J., Li L., Kabbinavar F., Ichinose Y. (2010). Vandetanib plus docetaxel versus docetaxel as second-line treatment for patients with advanced non-small-cell lung cancer (zodiac): A double-blind, randomised, phase 3 trial. Lancet Oncol..

[63] Dahlberg S.E., Sandler A.B., Brahmer J.R., Schiller J.H., Johnson D.H. (2010). Clinical course of advanced non-small-cell lung cancer patients experiencing hypertension during treatment with bevacizumab in combination with carboplatin and paclitaxel on ecog 4599. J. Clin. Oncol..

[64] Goodwin R., Ding K., Seymour L., LeMaître A., Arnold A., Shepherd F.A., Dediu M., Ciuleanu T., Fenton D., Zukin M. (2010). Treatment-emergent hypertension and outcomes in patients with advanced non-small-cell lung cancer receiving chemotherapy with or without the vascular endothelial growth factor receptor inhibitor cediranib: Ncic clinical trials group study br24. Ann. Oncol..

[65] Tsao M.S., Aviel-Ronen S., Ding K., Lau D., Liu N., Sakurada A., Whitehead M., Zhu C.Q., Livingston R., Johnson D.H. (2007). Prognostic and predictive importance of p53 and ras for adjuvant chemotherapy in non small-cell lung cancer. J. Clin. Oncol..

[66] Hallberg B., Palmer R.H. (2010). Crizotinib—Latest champion in the cancer wars?. N. Engl. J. Med..

[67] Kwak E.L., Bang Y.J., Camidge D.R., Shaw A.T., Solomon B., Maki R.G., Ou S.H., Dezube B.J., Jänne P.A., Costa D.B. (2010). Anaplastic lymphoma kinase inhibition in non–small-cell lung cancer. N. Engl. J. Med..

[68] Addison C.L., Ding K., Zhao H., Le Maître A., Goss G.D., Seymour L., Tsao M.-S., Shepherd F.A., Bradbury P.A. (2010). Plasma transforming growth factor α and amphiregulin protein levels in ncic clinical trials group br.21. J. Clin. Oncol..

[69] Karp D.D., Paz-Ares L.G., Novello S., Haluska P., Garland L., Cardenal F., Blakely L.J., Eisenberg P.D., Langer C.J., Blumenschein G. (2009). Phase ii study of the anti-insulin-like growth factor type 1 receptor antibody cp-751,871 in combination with paclitaxel and carboplatin in previously untreated, locally advanced, or metastatic non-small-cell lung cancer. J. Clin. Oncol..

[70] Jassem J., Langer C.J., Karp D.D., Mok T., Benner R.J., Green S.J., Park K., Novello S., Strausz J., Gualberto A. (2010). Randomized, open label, phase iii trial of figitumumab in combination with paclitaxel and carboplatin versus paclitaxel and carboplatin in patients with non-small cell lung cancer (NSCLC). ASCO Meet. Abstr..

[71] Kasahara K., Arao T., Sakai K., Matsumoto K., Sakai A., Kimura H., Sone T., Horiike A., Nishio M., Ohira T. (2010). Impact of serum hepatocyte growth factor on treatment response to epidermal growth factor receptor tyrosine kinase inhibitors in patients with non-small cell lung adenocarcinoma. Clin. Cancer Res..

[72] Spigel D.R., Ervin T.J., Ramlau R., Daniel D.B., Goldschmidt J.H., Blumenschein G.R., Krzakowski M.J., Robinet G., Clement-Duchene C., Barlesi F. (2011). Final efficacy results from oam4558g, a randomized phase ii study evaluating metmab or placebo in combination with erlotinib in advanced NSCLC. ASCO Meet. Abstr..

[73] Felip E., Ranson M., Cedres S., Brewster M., Mcnally V., Venturi M., Passioukov A., Ross G., Galdermans D. (2010). Biomarker analyses from a phase l study of pertuzumab combined with erlotinib in patients (pts) with non-small cell lung cancer (NSCLC). ASCO Meet. Abstr..

[74] Kumar S., Guleria R., Singh V., Bharti A.C., Mohan A., Das B.C. (2010). Plasma nucleosome levels might predict response to therapy in patients with advanced non-small-cell lung cancer. Clin. Lung Cancer.

[75] Zhu L.B., Xu Q., Hong C.Y., Yue Z., Zhang Y., Ye H.N., Yuan Y. (2010). Xpc gene intron 11 c/a polymorphism is a predictive biomarker for the sensitivity to np chemotherapy in patients with non-small cell lung cancer. Anti-Cancer Drugs.

[76] Chen H.Y., Yu S.L., Chen C.H., Chang G.C., Chen C.Y., Yuan A., Cheng C.L., Wang C.H., Terng H.J., Kao S.F. (2007). A five-gene signature and clinical outcome in non-small-cell lung cancer. N. Engl. J. Med..

[77] Park S., Holmes-Tisch A.J., Cho E.Y., Shim Y.M., Kim J., Kim H.S., Lee J., Park Y.H., Ahn J.S., Park K. (2009). Discordance of molecular biomarkers associated with epidermal growth factor receptor pathway between primary tumors and lymph node metastasis in non-small cell lung cancer. J. Thorac. Oncol..

[78] Cortot A.B., Italiano A., Burel-Vandenbos F., Martel-Planche G., Hainaut P. (2010). Kras mutation status in primary nonsmall cell lung cancer and matched metastases. Cancer.

[79] Vollebergh M.A., Kappers I., Klomp H.M., Buning-Kager J.C., Korse C.M., Hauptmann M., de Visser K.E., van den Heuvel M.M., Linn S.C. (2010). Ligands of epidermal growth factor receptor and the insulin-like growth factor family as serum biomarkers for response to epidermal growth factor receptor inhibitors in patients with advanced non-small cell lung cancer. J. Thorac. Oncol..

[80] Zalcman G., Levallet G., Bergot E., Antoine M., Creveuil C., Brambilla E., Dumontet C., Morin F., Depierre A., Milleron B. (2009). Evaluation of class iii beta-tubulin (btubiii) expression as a prognostic marker in patients with resectable non-small cell lung cancer (nsclc) treated by perioperative chemotherapy (ct) in the phase iii trial ifct-0002. J. Clin. Oncol..

[81] Herbst R.S., Blumenschein G.R., Kim E.S., Lee J., Tsao A.S., Alden C.M., Liu S., Stewart D.J., Wistuba I.I., Hong W.K. (2010). Sorafenib treatment efficacy and kras biomarker status in the biomarker-integrated approaches of targeted therapy for lung cancer elimination (BATTLE) trial. ASCO Meet. Abstr..

[82] Shepherd F.A., Tsao M.S. (2010). Epidermal growth factor receptor biomarkers in non-small-cell lung cancer: A riddle, wrapped in a mystery, inside an enigma. J. Clin. Oncol..

